# Development of a fluorescence-based cellular apoptosis reporter

**DOI:** 10.1088/2050-6120/aae6f8

**Published:** 2018-10-24

**Authors:** Lucy A Balderstone, John C Dawson, Arkadiusz Welman, Alan Serrels, Stephen R Wedge, Valerie G Brunton

**Affiliations:** 1 Cancer Research UK Edinburgh Centre, Institute of Genetics & Molecular Medicine, University of Edinburgh, Crewe Road South, Edinburgh, EH4 2XR, United Kingdom; 2 Northern Institute for Cancer Research, Newcastle University, Newcastle upon Tyne, NE2 4HH, United Kingdom; v.brunton@ed.ac.uk

**Keywords:** apoptosis, fluorescent probe, caspase

## Abstract

Evasion of apoptosis is a hallmark of human cancer, and a desired endpoint of many anticancer agents is the induction of cell death. With the heterogeneity of cancer becoming increasingly apparent, to understand drug mechanisms of action and identify combination therapies in cell populations, the development of tools to assess drug effects at the single cell level is a necessity for future preclinical drug development. Herein we describe the development of pCasFSwitch, a genetically encoded reporter construct designed to identify cells undergoing caspase-3 mediated apoptosis, by a translocation of a GFP signal from the cell membrane into the nucleus. Anticipated cellular distribution was demonstrated by use of confocal microscopy and cleavage by caspase-3 was shown to be required for the translocation of the GFP signal seen in apoptotic cells. Quantification of apoptosis using the construct revealed similar levels to that obtained with a commercially available apoptosis imaging agent (22.6% versus 20.3%). Moreover, we demonstrated its capacity for use in a high-throughput setting making it a powerful tool for drug development pipelines.

## 1. Introduction

Advances in fluorescent probe technology has seen the generation of an abundance of fluorescent probes or biosensors, which can be used to monitor complex cellular processes at a molecular level, such as small signaling molecule dynamics, protein-protein interactions and enzyme activation. Engineered fluorescent probes are extremely powerful tools for elucidating molecular drug mechanisms, and with the development of a wealth of fluorophores this allows multiple events to be monitored simultaneously at the single cell level. At a time when the heterogeneity of cancer is becoming increasingly apparent, the need for evaluation of drug effects at this level is even more pressing, aiding the visualization of differential treatment responses within cell populations.

Apoptosis (or programmed cell death) is an essential process required during embryogenesis and for the maintenance of normal tissue homeostasis. The evasion of cell death or apoptosis is also a hallmark of human cancer [1] with the desired endpoint of traditional cytotoxics and many molecularly targeted agents being the induction of cell death [2, 3]. Establishing a convenient and robust measurement of apoptosis at the single cell level in response to drug treatments would be a useful tool in preclinical drug development as we try and understand the complexities of drug mechanism of action and combination therapies. Caspases are a large family of cysteine proteases that play a central role in mediating the apoptotic response. To date, 11 human caspases have been identified: caspases-1-10 and -14 [4, 5]. Some caspases have evolved to link upstream signaling pathways to initiate apoptosis, while others execute the steps of cell death. Based on this function classification, caspases are classed as initiators (caspases- 1, -2, -4, -5, -8, -9, -10, -11, -12) or executioners (caspases-3, -6 and -7). All caspases are synthesized as inactive zymogens whose activation is mediated by either a series of cleavage events or allosteric conformational changes [6, 7]. To enable the specific evaluation of apoptotic cell death attention is being focused on the use of caspase activation as the target for fluorescent imaging agents. Specifically, caspase-3 is considered to be the central effector caspase since it is activated during the early stages of apoptosis during both the intrinsic and extrinsic caspase signaling cascade [8]. Activated caspase-3 recognizes a sequence of four amino acids (DEVD) in a number of target proteins such as cytokeratins and poly (ADP-ribose) polymerase (PARP) whose cleavage leads to cell death. To date caspase-3 activation as an indicator of apoptosis has been measured using a number of different approaches. One approach has been the use of fluorescence resonance energy transfer (FRET) pairs linked by caspase cleavage sequences such as DEVD, which allows measurement of caspase activation in single cells [9, 10]. Other methodologies have used either loss of fluorescence quenching, switching-on of fluorescence or translocation of fluorescent proteins following caspase cleavage to monitor apoptosis [11–16]. Here we describe the generation of a novel genetically encoded caspase-3 reporter which is based on the translocation of GFP from the plasma membrane to the nucleus following cleavage of the DEVDG peptide sequence allowing quantitative measurements of apoptosis at the single cell level.

## 2. Experimental

2.

### 2.1. Generation of constructs

pEGFP-N1 (https://addgene.org/vector-database/2491/) was digested with Xho1 and BamH1 and ligated with forward (F) and reverse (R) single-stranded synthetic NLS oligonucleotides (table 1). Following transformation of competent *E. coli* and DNA purification, positive clones containing the NLS insert were identified by Xho1 and BamH1 digestion and agarose-gel electrophoresis. The above process was repeated to sequentially insert the PLS oligonucleotides (using BsrG1 and Not1) and the DEVDG oligonucleotides (BsrG1 and EcoRV). Resulting pNGD6 and pNGDH constructs were Sanger sequenced using primers 5′GTCGCCGTCCAGCTCGACCAG3′ and 5′CATGGTCCTGCTGGAGTTCGTG3′ respectively. For generation of pNGNH and pNGNH mutants, site directed mutagenesis was carried out using F (5′ GTGACGAGGTCAACGGTACCTCAGTC 3′) and R (5′ GACTGAGGTACCGTTGACCTCGTCAC 3′) primers and Sanger sequenced using primer 5′ TGAACTTCAAGATCCGCCAC 3′ to ensure mutation of the construct. NGD6 and NGN6 were then introduced into pBABEpuro retroviral vector. Blunt-end PCR products were generated by combining 10 ng of construct with 100 ng F (5′ TACGTAATGGATCCAAAAAAG 3′) and R (5′ GCGGCCGCTTACATAATTAC 3′) primers in PfuUltra Hotstart PCR Master Mix (Agilent Technologies). Purified DNA was cloned into TOPO using the Zero Blunt Topo PCR cloning kit (Invitrogen). DNA and pBABE vector was digested using SnaB1 and EcoR1 restriction endonucleases. Inserts digested from pCRII-Blunt-Topo were purified alongside the digested pBABE using QIAquick Gel extraction kit. Insert and vector were ligated using Rapid DNA ligation kit (Roche) before proceeding to bacterial transformation, amplification, and extraction using Qiagen Plasmid Plus Maxi Kit. Constructs were Sanger sequenced using primers 5′ TACGGCGTGCAGTGCTTCAG 3′, 5′CTGAAGCACTGCACGCCGTA3′, 5′TGAACTTCAAGATCCGCCAC3′, 5′GTGGCGGATCTTGAAGTTCA3′, 5′AAGGGCGAGGAGCTGTTCAC3′, 5′GTGAACAGCTCCTCGCCCTT3′, 5′ATCACTCTCGGCATGGACGA3′, 5′TCGTCCATGCCGAGAGTGAT3′.

**Table 1.** Oligonucleotide sequences. Oligonucleotide nomenclature and sequences used for the generation of the in-house apoptosis imaging agent. F and R denote forward and reverse oligonucleotide respectively. Oligonucleotides were dissolved in 100 *μ*l of dH_2_0. All oligonucleotides were purchased from Invitrogen.

Name	F/R	Sequence 5′—3′
2NLS	F	TCGAGATGGATCCAAAAAAGAAGAGAAAGGTAGATCCAAAAAAGAAGAGAAAGGTAGGATCCACCGGATCTAGA
	R	GATCTCTAGATCCGGTGGATCCTACCTTTCTCTTCTTTTTTGGATCTACCTTTCTCTTCTTTTTTGGATCCATC
3NLS	F	TCGAGATGGATCCAAAAAAGAAGAGAAAGGTAGATCCAAAAAAGAAGAGAAAGGTAGATCCAAAAAAGAAGAGAAAGGTAGGATCCACCGGATCTAGA
	R	GATCTCTAGATCCGGTGGATCCTACCTTTCTCTTCTTTTTTGGATCTACCTTTCTCTTCTTTTTTGGATCTACCTTTCTCTTCTTTTTTGGATCCATC
DEVDG	F	GTACAAGGGAGGCAACAGCGGTGACGAGGTCGACGGTACCTCAGTCGCCACCGGAAGCGAT
	R	ATCGCTTCCGGTGGCGACTGAGGTACCGTCGACCTCGTCACCGCTGTTGCCTCCCTT
HRPLS	F	GTACAAGGATATCAAGCTGAACCCTCCTGATGAGAGTGGCCCCGGCTGCATGAGCTGCAAGTGTGTGCTCTCCTGAGC
	R	GGCCGCTCAGGAGAGCACACACTTGCAGCTCATGCAGCCGGGGCCACTCTCATCAGGAGGGTTCAGCTTGATATCCTT
6KPLS	F	GTACAAGGATATCAAAAAGAAGAAAAAGAAGTGTGTAATTATGTAAGC
	R	GGCCGCTTACATAATTACACACTTCTTTTTCTTCTTTTTGATATCCTT

### 2.2. Cell culture and transfection

Murine mammary carcinoma-derived 4T1 cells were cultured in DMEM supplemented with 10% heat-inactivated FCS and 2 mM L-Glutamine while murine squamous cell carcinoma SCC cells were cultured in GMEM, 10% heat-inactivated FCS, 2 mM L-Glutamine, 1 mM Sodium Pyruvate, Non-essential Amino acids, Vitamins and 0.5 mg ml^−1^ Hygromycin. Both were maintained in a humidified atmosphere at 37 °C and 5% CO_2_. 4T1 cells were transiently transfected with pNGD6/pNGN6 and pNGDH/pNGNH using Lipofectamine 2000 reagent and left at 37 °C and 5% CO_2_ for 24 h prior to experiments. SCC cells were stably transduced with the constructs pBABE-NGD6/NGN6 using a retroviral transduction method. Phoenix ecotropic (Phoenix eco) cells were seeded 36 h prior to Lipofectamine transfection with the retroviral constructs. The transfection medium was removed 24 h later and replaced with 5 ml of fresh media supplemented with 20% FBS. After 24 h the viral supernatant was collected, filtered through a 0.45 *μ*m membrane, and added to target cells in the presence of 4 *μ*g ml^−1^ polybrene. This was repeated a further 2 times. Cells stably expressing the construct were selected with 1 *μ*g ml^−1^ puromycin and the brightest cells were single cell FACS on a BD FACSAriaTM II (BD Biosciences) machine. Cells were maintained in media containing 1 *μ*g ml^−1^ puromycin.

### 2.3. Confocal microscopy analysis

Collagen coated coverslips in the bottom of 12 well plates were seeded with cells at a density of 1 × 10^4^ in 1 ml growth medium. For assessment of cleavage of the final construct, 24 h post-seeding, 4T1 cells were treated with 4 *μ*M doxorubicin (Sigma) and SCC cells with 250 nM staurosporine (Sigma). After 24 h, cells were fixed by the addition of 1 ml 8% paraformaldehyde for 20 min, washed once in PBS, incubated with Hoechst (1:5000 in PBS) for 10 min at room temperature, rinsed two further times in PBS and mounted using Vectashield mounting medium (Vector Laboratories Ltd). Alternatively, if immunofluorescence of cleaved caspase-3 (Cell Signaling) was required, following the first PBS wash, cells were incubated in IF blocking buffer (PBS containing 1% BSA and 0.2% Triton-X100) for 30 min. Primary antibody, diluted 1:400 in IF blocking buffer, was added for 1 h. Cells were washed once in IF blocking buffer and incubated in the buffer for 30 min. Alexa fluor 594 secondary antibody and Hoechst (1:5000, Invitrogen) diluted in IF blocking buffer were added to the wells for 45 min in the dark. Cells were washed twice in PBS and mounted. Cells were viewed using an Olympus FV1000 confocal microscope. For quantification of apoptosis using the construct the number of cells with nuclear GFP were counted manually in five low magnification images and plotted as a function of the total number of cells identified by Hoechst staining. GFP positive nuclei were identified by co-localization with a Hoechst signal. All images were scored blinded. Each experiment was performed in triplicate and independently repeated three times.

### 2.4. Western blot analysis

Cells to be analyzed by western blotting were seeded at an appropriate density in 100 mm dishes and treated with doxorubicin or staurosporine as indicated. For assessment of probe cleavage SCC and SCC NGD6 cells were left untreated, or SCC NGD6 cells were exposed to 250 nM staurosporine for 24 h: lysates from untreated SCC NGD6 cells were incubated with recombinant Caspase-3 or Cathepsin B (Millipore) for given time points. Cell lysis was carried out with RIPA buffer and protein concentration of the supernatants determined using a Micro BCA^TM^ protein assay kit (Thermo Scientific). Lysates were separated using 4%–12% SDS-PAGE gels and electrophoretically transferred onto a nitrocellulose membrane. The membrane was probed with the desired primary antibody; Cleaved caspase-3 (1:1000, Cell Signaling), PARP (1:1000, Cell Signaling), Anti-Tag (CGY)FP (1:10000 Evrogen) or *β*-actin (1:1000, Sigma). Membranes were subsequently incubated with the relevant Licor Anti-mouse 680 or Anti-rabbit 800 secondary antibody and visualized using the LI-COR Odyssey SA system.

### 2.5. NucView apoptosis assay

SCC cells were seeded at a density of 1000 cells/well into Nunc 96 well optical bottom plates (Thermo Scientific) with 1 *μ*M NucView (Biotium). After 24 h cells were exposed to increasing concentrations of staurosporine ranging from 7 nM to 1 *μ*M. At 16, 24, 36 or 48 h post drug addition, nuclei were stained with Hoechst (1:5000) for 10 min at 37 °C. Plates were imaged with the Olympus ScanR microscope using excitation and emission filter cubes for DAPI and FITC and brightfield using a 20× objective. Per well 9 images were taken using a 3 by 3 grid with 800 *μ*m spacing and field of view 433 by 330 *μ*m. The DAPI channel was used for autofocus. Images were analysed using the ScanR analysis software according to the manufacturer’s instructions. Briefly, the number of nuclei and NucView positive objects were identified by setting a user defined local threshold in the DAPI and FITC channels, respectively. The threshold was determined from inspection and testing on several images from both untreated and treated cells prior to analysis of the whole experiment. Finally, the percentage NucView positive nuclei (where nuclear objects also had a NucView object) was calculated in Microsoft Excel.

### 2.6. ImageXpress high-throughput assay

SCC cells were seeded in Nunc 96 well optical bottom plates and cultured for 24 h. Alternate columns were treated with either DMSO (0.1% final concentration) or 1000 nM staurosporine and cells cultured for a further 24 h. Cell nuclei were labelled with Hoechst (2 *μ*g ml^−1^; Invitrogen) and plates were imaged on an ImageXpress Micro XLS microscope (Molecular Devices) using the DAPI and FITC filters. Images were analyzed using CellProfiler [17]: briefly, positive nuclei were identified using an Otsu threshold on the Hoechst channel to identify individual cells. For NGD6 and NGN6 expressing cells using the nucleus as a seed, a secondary object (the whole cell) was derived from the FITC channel and then identified by using the ‘propagation’ function. The nuclear object was subtracted from the whole cell to give the cell body which represents the non-nuclear signal. Finally, the intensity of the GFP probe in the nucleus and the cell body objects was measured. Well averages were calculated from single cell data of cell body GFP intensity and normalized as percentage of DMSO controls. SCC cells treated with NucView (identified as above) were classified as NucView positive or negative. Nucview positive nuclei were identified by setting an Otsu threshold in the FITC channel to generate a NucView mask. Nuclear objects that also had an associated NucView object were classed as apoptotic and expressed as a percentage of the population. Data was handled in Excel and GraphPad Prism. A D’Agostino and Pearson omnibus K2 test was used to confirm a normal distribution of the data (GraphPad Prism). Assay robustness was tested using a Z-factor analysis [18].

## 3. Results and discussion

### 3.1. Generation of an apoptosis reporter construct named pCasFswitch

**Figure 1.**
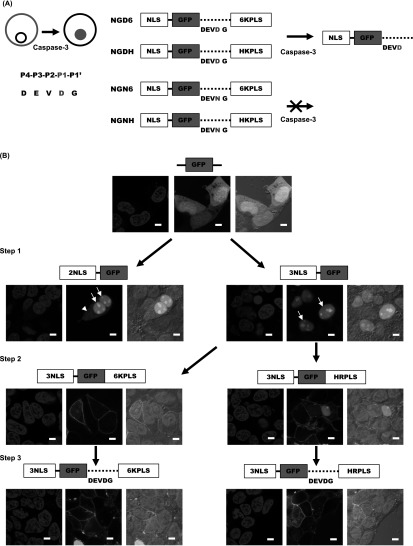
Design and generation of pCasFSwitch. (A) pCasFSwitch is a fluorescent reporter construct designed to identify cells undergoing caspase-3 mediated apoptosis by a switch in a GFP signal from the plasma membrane of the cell to the nucleus. Schematic representations of the constructs showing the caspase-3 cleavage site in red. Probe nomenclature shown beside each schematic. (B) pCasFSwitch was constructed from pEGFP-N1. Step 1: insertion of two or three nuclear localization sequences (NLS) at the N-terminal of GFP, to generate p2NLS-GFP and p3NLS-GFP respectively. White arrows indicate nuclear GFP, and white arrowheads show cytosolic GFP. Step 2: insertion of plasma membrane localization sequences (PLS) at the C-terminal of GFP, using the hexalysine stretch employed by K-Ras4B, or the hypervariable domain of H-Ras, yielding the intermediate constructs pNLS-GFP-6KPLS and pNLS-GFP-HRPLS respectively. Step3: insertion of the caspase-3 cleavage domain between GFP and the PLS to generate the final constructs, pNLS-GFP-DEVDG-6KPLS (NGD6) and pNLS-GFP-DEVDG-HRPLS (NGDH). Functionality testing for each of the constructs was carried out by transient transfection into HEK293T cells and analysis by confocal microscopy. Representative images for each of the intermediate constructs is shown below the appropriate construct schematic. Blue = nuclei stained with Hoechst; Green = cellular distribution of the GFP construct. Merge = overlay of blue and green channels superimposed on DIC image. Scale bars = 10 *μ*m.

pCasFswitch is a fluorescent based reporter, designed to reveal cells undergoing caspase-3 mediated apoptosis. The structure of the reporter is shown in figure 1(A). It incorporates a GFP sequence flanked at the N-terminus by a nuclear localization sequence (NLS), and at the C-terminus by a plasma membrane localization sequence (PLS) separated from the GFP by the caspase-3 cleavage domain (DEVDG). The probe is designed on the assumption that the plasma membrane targeting sequence is stronger than the nuclear localization sequence. So, the probe will be located at the plasma membrane via the PLS, and upon cleavage by caspase-3 at the DEVDG domain (between D and G), the GFP-NLS will accumulate in the nucleus. The construct was constructed by the stepwise modification of pEGFP-N1 through ligation of synthetic oligonucleotides. The first step involved the insertion of NLS at the N-terminal of GFP, one containing two NLS repeats, and the other containing three repeats, to generate p2NLS-GFP and p3NLS-GFP respectively. The NLS sequence was based on that of the Simian Vacuolating Virus 40 (SV40) Large T-antigen [19]. Fluorescence images of cells transfected with these intermediate constructs revealed that with two NLS repeats, the reporter was localized both in the nucleus and the cytoplasm. On the contrary with three repeats the GFP signal was strongly nuclear, and this construct was therefore utilized for subsequent modification (figure 1(B)). The second step incorporated one of two PLS at the C-terminal of GFP: one took advantage of the hexalysine stretch employed by K-Ras4B, and the other was based on the hypervariable domain of H-Ras [20, 21], yielding the intermediate constructs pNLS-GFP-6KPLS and pNLS-GFP-HRPLS respectively. The addition of both PLS resulted in the translocation of the majority of the GFP to the plasma membrane, subsequently both constructs were completed by the addition of the caspase-3 cleavage domain between the GFP and PLS domains to yield two reporter constructs for validation; pNLS-GFP-DEVDG-6KPLS and pNLS-GFP-DEVDG-HRPLS (referred to as NGD6 and NGDH from here on) (figure 1(B)). Addition of the cleavage domain did not affect the cellular distribution of GFP (figure 1(B)). We also generated non-cleavable reporters in which the aspartic acid at the P_1_ position was changed to an asparagine residue yielding the control constructs NGN6 and NGNH ([22–24], figure 1(A)).

### 3.2. Translocation of pCasFswitch to the nucleus following induction of apoptosis

**Figure 2.**
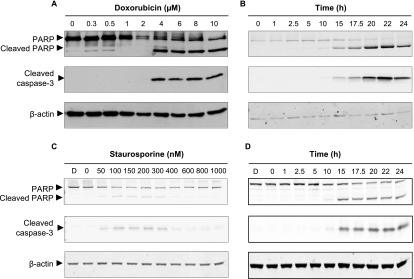
Activation of caspase-3 in 4T1 and SCC cells. (A) Cell lysates from 4T1 cells treated with increasing concentrations of doxorubicin for 24 h, or (B) 4T1 cells treated with 4 *μ*M doxorubicin for given time periods, or (C) SCC cells treated with increasing concentrations of staursporine for 24 h, or (D) SCC cells treated with 250 nM staurosporine for given time periods, were subjected to western blot analysis using cleaved caspase-3 and PARP antibodies. All membranes were stripped and re-probed with *β*-actin as a loading control.

In order to validate the constructs, conditions that activate the target of the probe (caspase-3) in cells needed to be determined. 4T1 cells are derived from a mouse mammary tumor and were treated with doxorubicin which is a drug widely utilized for the treatment of breast cancer and known to induce apoptosis [25]. To determine the doxorubicin concentration required to activate apoptosis, 4T1 cells were treated with increasing concentrations of doxorubicin for 24 h and analyzed by western blotting. Western blot analysis of cell lysates from the adherent population showed that cleavage of caspase-3, and its downstream substrate poly(ADP-ribose) polymerase (PARP) [8], was achieved at concentrations of 4 *μ*M doxorubicin and above (figure 2(A)). The timescale of caspase-3 activation was then assessed by exposing cells to 4 *μ*M doxorubicin for specified time periods. Western blotting showed that 15 h was the first time point at which cleavage of caspase-3 and PARP had occurred (figure 2(B)). So, doxorubicin was utilized *in vitro* at a concentration of 4 *μ*M for 15 h to activate caspase-3 in adhered cells to validate the apoptosis imaging agents. To evaluate the generality of the approach an additional cancer cell line and apoptosis-inducing agent was employed in this initial validation. The mouse squamous cell carcinoma (SCC) cell line [26]) was treated with staurosporine, a drug that is widely utilized to induce apoptosis. SCC cells were exposed to increasing concentrations of staurosporine and western blot analysis showed that cleavage of caspase-3 was achieved using concentrations of 50 nM staurosporine and above (figure 2(C)). Activation of caspase-3 was confirmed with these concentrations by the cleavage of its downstream substrate PARP (figure 2(C)). Exposure of the cells to 250 nM staurosporine for specified time periods showed that the cleavage of caspase-3 and PARP could be detected at 15 h post exposure (figure 2(D)). So, staurosporine was used *in vitro* at a concentration of 250 nM for a minimum of 15 h.

**Figure 3.**
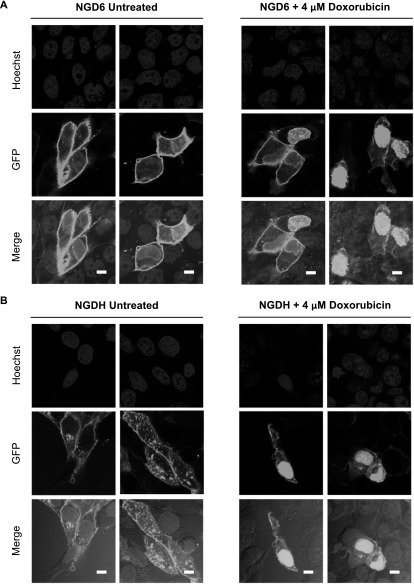
Cellular distribution of NGD6 and NGDH in 4T1 cells. Representative confocal images of (A) 4T1 NGD6 cells and (B) 4T1 NGDH cells untreated or treated with 4 *μ*M doxorubicin for 15 h. Blue = nuclei stained with Hoechst; Green = cellular distribution of GFP construct; Merge = overlay of channels. Scale bars = 10 *μ*m.

**Figure 4.**
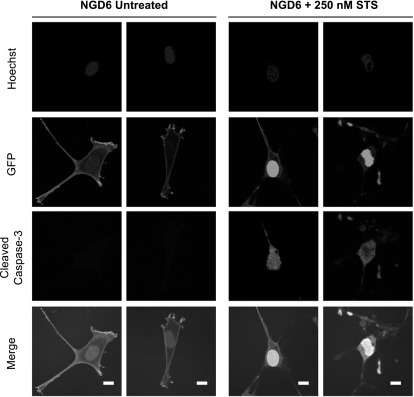
Cellular distribution of NGD6 in SCC cell line. Representative confocal images of SCC NGD6 cells untreated or treated with 250 nM staurosporine for 24 h (STS). Blue = nuclei stained with Hoechst; Green = cellular distribution of GFP construct; Red = Cleaved caspase-3 immunofluorescence; Merge = overlay of channels. Scale bars = 10 *μ*m.

Once general parameters to activate caspase-3 in the 4T1 and SCC cells were established we then looked at the localization of pCasFswitch following induction of apoptosis. In the majority of untreated 4T1 cells expressing NGD6, GFP was localized predominantly at the plasma membrane. A few cells with nuclear GFP were evident, however, upon doxorubicin treatment the number of cells expressing nuclear GFP increased (figure 3(A)). Similar observations were made in 4T1 cells transfected with the NGDH construct although there were low levels of cytoplasmic staining in both untreated and treated cells (figure 3(B)). The NGD6 construct was therefore taken forward for further analysis in SCC cells following stable expression of the constructs. Fluorescence images of untreated SCC NGD6 expressing cells showed GFP was localized predominantly at the plasma membrane, and very occasionally, a few cells with some nuclear GFP were evident (figure 4). Upon incubation of the cells with 250 nM staurosporine for 24 h, the number of cells that contained nuclear GFP increased (figure 4). Use of a cleaved caspase-3 antibody confirmed that cells with nuclear GFP were expressing cleaved caspase-3 (figure 4, bottom panels).

### 3.3. pCasFswitch is cleaved by caspase-3

**Figure 5.**
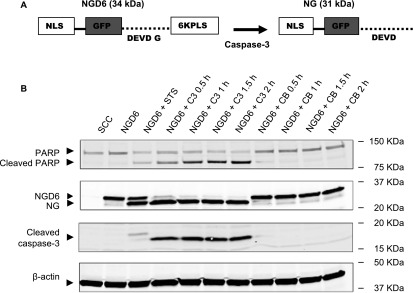
Cleavage analysis of NGD6 in SCC cell line. (A) Schematic representation of caspase-3 mediated cleavage of NGD6 showing the un-cleaved and cleaved structures. Probe nomenclature and molecular weight shown beside each schematic. (B) Lysates from; SCC cells, SCC NGD6 cells left untreated or treated with 250 nM staurosporine for 24 h (STS), and untreated SCC NGD6 cells incubated with recombinant caspase-3 (C3) or Cathepsin B (CB) for given time periods (as indicated), were subjected to western blot analysis using GFP, cleaved caspase-3 and PARP antibodies. All membranes were stripped and re-probed with *β*-actin as a loading control.

Characterization of pCasFswitch cleavage by recombinant caspase-3 was carried out. SCC NGD6 cells were left untreated, or treated with 250 nM staurosporine for 24 h, and lysates subjected to western blot analysis using a GFP antibody. NGD6 expressing cells would be expected to generate two GFP bands on a western blot; one for the un-cleaved probe at 34 kDa, and the other for the cleaved probe at 31 kDa (figure 5(A)). Only a 34 kDa band was evident in untreated cells corresponding to the un-cleaved probe, the level of which decreased upon staurosporine treatment, with the concomitant appearance of a 31 kDa band corresponding to the cleaved probe (figure 5(B)). The absence of bands upon exposure of SCC lysates to the GFP antibody confirmed the presence of bands in SCC NGD6 lysates were specific to the construct. Incubation of SCC NGD6 lysates with recombinant caspase-3 (C3) for various times, but not Cathepsin B (CB), resulted in the appearance of the 31 kDa cleavage product, and confirmed the construct was cleaved by caspase-3 and could not be cleaved by other members of the cysteine protease family of proteins. Probing of the membranes with the cleaved caspase-3 antibody confirmed all construct cleavage was occurring with the presence of active caspase-3, with differences in the molecular weight of the bands detected corresponding to the native and recombinant protein respectively. The observation of PARP cleavage under these conditions further confirmed the activity of the recombinant caspase-3 in the lysates (figure 5(B)).

### 3.4. Cleavage of pCasFswitch is required for translocation to the nucleus

**Figure 6.**
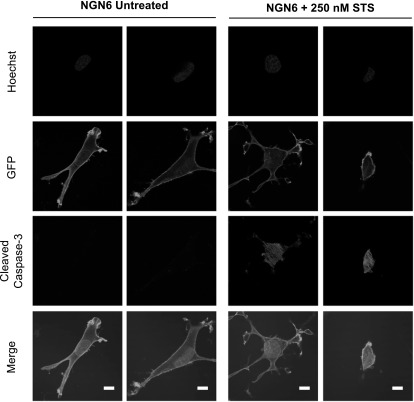
Cellular distribution of NGN6 in SCC cell line. Representative confocal images of SCC NGN6 cells untreated or treated with 250 nM staurosporine for 24 h (STS). Blue = nuclei stained with Hoechst; Green = cellular distribution of GFP construct; Red = Cleaved caspase-3 immunofluorescence; Merge = overlay of both channels. Scale bars = 10 *μ*m.

To determine whether caspase-3 dependent cleavage of pCasFswitch was required for its translocation to the nucleus we looked at localization of the non-cleavable NGN6 reporter (figure 1(A)). In SCC NGN6 expressing cells, GFP was localized at the plasma membrane in untreated cells, and no translocation of GFP to the nucleus was evident in staurosporine treated cells (figure 6). Caspase-3 activation in the staurosporine treated cells was confirmed by the presence of cleaved caspase-3 in the cells detected by immunofluorescence (figure 6).

### 3.5. Quantitative analysis of apoptosis using pCasFswitch

**Figure 7.**
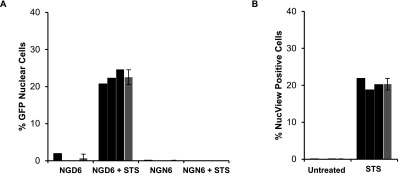
Quantification of staurosporine mediated apoptosis using SCC NGD6 and NGN6 cells. (A) Percentage of cells with nuclear GFP calculated for the constructs and treatment conditions indicated. STS = treatment with 250 nM staurosporine for 24 h. Black bars represent the mean of one experiment performed in triplicate, green bars represent the mean of three independent experiments +/−SD. (B) Percentage NucView positive cells calculated for the treatment conditions indicated. STS = treatment with 250 nM staurosporine for 24 h. Black bars represent the mean of one experiment performed in triplicate, green bars represent the mean of three independent experiments +/−S.D.

To provide a quantitative read-out of apoptosis SCC NGD6 and NGN6 expressing cells were either left untreated or treated with 250 nM staurosporine for 24 h. The number of cells in which GFP was present in the nucleus was then calculated in five low magnification images. The number of GFP nuclear cells were counted and plotted as a function of the total number of cells identified by Hoechst staining. Each experiment was performed in triplicate, to allow identification of inter well anomalies, and the mean calculated. Nuclear GFP fluorescence was evident in 22.6% of staurosporine treated SCC NGD6 cells, and 0% of staurosporine treated cells expressing the non-cleavable NGN6 construct (figure 7(A)). This is in good agreement (p = 0.1966 by Student’s *t* test) with the mean of 20.3% apoptotic cells identified using NucView over three independent experiments (figure 7(B)). NucView employs a fluorogenic enzyme substrate design in which a nucleic acid dye is attached to the caspase-3/7 substrate peptide sequence DEVD. In this linked state, the dye is unable to bind DNA and remains non-fluorescent. Once the substrate becomes cleaved, the NucView 488 DNA dye can migrate to the nucleus, and upon binding DNA yields a bright green fluorescence [27].

**Figure 8.**
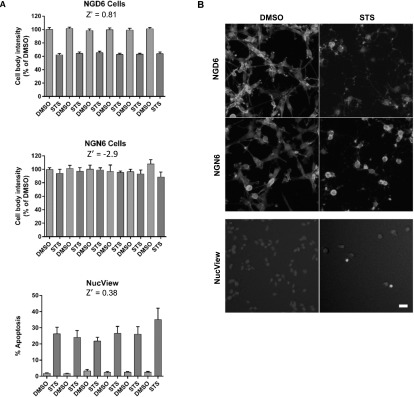
Quantification of staurosporine mediated apoptosis using ImageXpress. (A) SCC NGD6 or SCC NGN6 cells treated with staurosporine (STS) for 24 h. SCC cells incubated with NucView apoptosis reagent were included as a comparison. Graphs represent column averages from each 96 well plate ± standard deviation. A Z-factor (Z’) analysis was performed on each plate. (B) Representative images of SCC NGD6 and SCC NGN6 cells or NucView treated SCC cells +/−staurosporine for 24 h. Blue = nuclei stained with Hoechst; Green = cellular distribution of GFP construct or NucView. Scale bar = 20 *μ*m.

To further validate the probe for high-throughput analysis we used the ImageXpress high-content analysis system widely used in high-throughput drug screening pipelines [28]. Analysis of multiple 96-well plates demonstrated excellent inter-plate reproducibility (figure 8(A)) and comparison of the quantitative analysis of apoptosis using the NGD6 reporter and NucView showed good agreement between the two approaches. Furthermore, calculation of the Z-factor for the NGD6 reporter assay, which is used in high-throughput screening as a measure of statistical effect size was excellent (Z = 0.81) (figure 8(A)).

## 4. Conclusions

In conclusion, we have developed a novel genetically encoded fluorescent apoptosis reporter construct designed to identify cells undergoing caspase-3 mediated apoptosis through translocation of a GFP construct from the cell membrane into the nucleus. Other apoptosis reporters, based on translocation of GFP to the nucleus following caspase cleavage have been generated. Bardet and co-workers used a caspase sensitive site from the caspase inhibitor Drosophila inhibitor of apoptosis protein 1 (DIAP1) rather than the DEVD sequence used in pCasFSwitch [11]. DIAP1 is cleaved by downstream effector caspases and was also shown to be an effective read-out of caspase activation. However, in untreated cells nuclear GFP was evident in all cells [11] and to overcome this limitation we incorporated a PLS which effectively excluded GFP from the nucleus (figure 1). Previous studies have also reported low signal-to-noise ratios due to significant retention of GFP in the cytoplasm of apoptotic cells [13–15]. This raised the possibility that the single NLS sequence used in these reporters was not sufficient for the robust nuclear localization of GFP upon caspase-dependent cleavage. Indeed, we show that the addition of 3 NLS sequences increased the nuclear localization of GFP as compared to a single NLS (figure 1). However, we did still see evidence of non-nuclear GFP and indeed within the cytoplasm of apoptotic cells. The high expression of pCasFSwitch in cells coupled with the tendency of GFP to distribute to the cytoplasm [15] may account for this distribution pattern. However, importantly we were able to demonstrate the utility of pCasFSwitch for higher throughput modalities providing robust quantitative measurements based on its translocation in apoptotic cells. We envisage that pCasFSwitch can provide a powerful preclinical drug development tool, enabling interrogation of drug effects in live single cells in real-time after a simple transfection providing a cheaper alternative to the use of agents such as NucView or antibody-based approaches. Nicholls and co-workers have developed a caspase activity reporter based on the use of a genetically encoded GFP fused to a peptide which quenches the fluorescence signal. Incorporation of a DEVD cleavage site allows removal of the quenching peptide upon caspase activation and results in increased GFP fluorescence and provides a more direct read-out of apoptosis that does not depend on nuclear translocation [12]. Direct comparison with pCasFSwitch would be required to determine whether this provides a more robust approach for use in high-throughput imaging systems. Previous studies have shown that imaging of apoptosis can be achieved based on direct visualization of nuclear fragmentation, that occurs during the apoptotic process, using cells expressing histone H2B-GFP in the nucleus [29, 30]. This provides a useful qualitative read-out of different stages of the apoptotic process in real-time although quantitative measurements for high-throughput assays would be difficult. Further developments could see the use of pCasFSwitch extended to the *in vivo* environment, allowing extrapolation of *in vitro* and *in vivo* data. Indeed, we have shown the utility of a genetically encoded photoactivatable two-color probe for real-time tracking of cells in *Drosophila* [19]. The use of intravital imaging to provide subcellular distribution of proteins in mouse models opens up the possibility of tracking apoptosis in real time *in vivo* [31].
